# Premature Activation of the HIV-1 Protease Is Influenced by Polymorphisms in the Hinge Region

**DOI:** 10.3390/v16060849

**Published:** 2024-05-26

**Authors:** Caroline O. Tabler, Sarah J. Wegman, Najwa Alhusaini, Nicole F. Lee, John C. Tilton

**Affiliations:** Center for Proteomics and Bioinformatics, Department of Nutrition, School of Medicine, Case Western Reserve University, Cleveland, OH 44106, USA; cot3@case.edu (C.O.T.); nsa29@case.edu (N.A.);

**Keywords:** HIV-1 protease, premature protease activation, drug resistance, protease inhibitor, flow virometry, nanoscale flow cytometry

## Abstract

HIV-1 protease inhibitors are an essential component of antiretroviral therapy. However, drug resistance is a pervasive issue motivating a persistent search for novel therapies. Recent reports found that when protease activates within the host cell’s cytosol, it facilitates the pyroptotic killing of infected cells. This has led to speculation that promoting protease activation, rather than inhibiting it, could help to eradicate infected cells and potentially cure HIV-1 infection. Here, we used a nanoscale flow cytometry-based assay to characterize protease resistance mutations and polymorphisms. We quantified protease activity, viral concentration, and premature protease activation and confirmed previous findings that major resistance mutations generally destabilize the protease structure. Intriguingly, we found evidence that common polymorphisms in the hinge domain of protease can influence its susceptibility to premature activation. This suggests that viral heterogeneity could pose a considerable challenge for therapeutic strategies aimed at inducing premature protease activation in the future.

## 1. Introduction

The HIV-1 protease is a 99-amino-acid aspartic protease that is essential for the generation of infectious virions. Protease is catalytically active as a homodimer and processes at least 11 unique cleavage sites in the viral Gag and GagPol polyproteins. Protease dimers are stabilized primarily by a β-sheet formed by its N- and C-termini, and dimerization begins when protease is still embedded within the GagPol polyprotein [1,2]. Protease activity regulates both fusion and post-fusion events. For example, the protease-mediated processing of Gag initiates the formation of a conical capsid shell that protects the viral genome from innate viral sensors and assists in transporting the genome to the cellular nucleus [3,4,5]. The processing of Gag also regulates viral fusion, resulting in the softening of the viral membrane and aggregation of the fusogenic envelope [6,7,8,9,10,11].

Protease, along with the viral enzymes reverse transcriptase and integrase, are the primary targets of HIV-1 antiretroviral therapy. As with all inhibitors used to treat HIV-1 infection, HIV can develop drug resistance mutations that render these drugs ineffective. Patients are typically treated with a combination of inhibitors targeting the integrase, reverse transcriptase, and/or protease so that multiple resistance mutations must develop for the treatment to fail. However, resistance mutations can still develop, especially when a strict dosing regimen is not carefully maintained.

A significant advantage of protease inhibitors is their relatively high genetic barrier to resistance. For example, a single amino acid substitution can cause treatment with reverse transcriptase inhibitors to fail, but four or more mutations are required for the same result to occur with most protease inhibitors [12,13,14,15]. Protease inhibitors are recommended prior to genotypic sequencing when the presence of inhibitor resistance mutations is unclear, specifically if a patient has a history of cabotegravir use as pre-exposure prophylaxis that could have selected for integrase resistance mutations [16]. Protease inhibitors are also beneficial for treatment-experienced patients that have developed resistance to other inhibitors, as well as in resource-limited areas where drug resistance mutations are common [17]. In addition, recently developed protease inhibitors like darunavir interact more extensively with protease and select for a unique set of resistance mutations, making them effective even after a virus becomes resistant to other protease inhibitors [18]. The World Health Organization estimates that at least 1.5 million people in low- and middle-income countries will be on a second-line regimen including a protease inhibitor by 2025 [19].

The Stanford Resistance Database recognizes at least 35 of the 99 amino acids in protease as being associated with protease inhibitor resistance [20,21]. Protease inhibitor resistance mutations are commonly classified as either major or accessory mutations. Major mutations directly interfere with inhibitor binding and typically develop first. However, major mutations often result in structural changes that impair protease function and harm viral replication [22,23]. Protein structural modeling indicates that major mutations generally destabilize the protease dimer, while accessory mutations either compensate for these replicative defects or incrementally contribute to drug resistance when combined with a major mutation [22,23,24]. Several highly variable protease polymorphisms have also been linked to inhibitor resistance [22,25]. However, by definition, polymorphic mutations occur commonly in treatment-naïve patients, and they are rarely categorized as resistance mutations [22].

In addition to protease inhibitors, a potential strategy for inhibiting viral replication is to manipulate the kinetics of protease activation. In a previous study, we demonstrated that protease activation typically occurs concurrently with viral budding from the host cell [26]. However, protease can also become active too soon, prior to viral budding, an occurrence known as premature protease activation. The processing of Gag and GagPol within the cytosol prevents functional viral particles from being formed [27,28]. Premature protease activation not only interferes with virus production but can also cause the infected cell to die. The HIV-1 protease can process cellular proteins including BCL-2 and procaspase 8, resulting in apoptosis, and the cellular inflammasome CARD8, which activates pyroptosis [29,30,31]. Because killing infected cells is a necessary step in curing HIV-1 infection, promoting premature protease activation is being investigated as a tool for specifically destroying infected cells. The non-nucleoside reverse transcriptase inhibitors efavirenz and rilpivirine have been shown to promote premature protease activation in vitro [31,32]. These drugs bind to reverse transcriptase within the GagPol polyprotein and cause it to multimerize in the host cytosol. As a result, the dimerization and activation of protease within GagPol are also facilitated. Despite showing promise in vitro, this strategy has proven difficult to replicate in vivo due to the high drug concentration required to promote premature protease activation [31].

Here, we compared some of the most common major and accessory protease resistance mutations based on their impacts on protease activity and premature protease activation using a novel nanoscale flow cytometry-based reporter assay. We also examined the replicative fitness of each virus using a reporter T cell line. We found that major mutations overwhelmingly reduced protease function, while accessory mutations could be subdivided based on their ability to either reduce or improve protease activity. The calculated change in thermodynamic protease stability caused by each mutation also correlated with its resulting impact on protease activation. We also tested a panel of patient-derived proteases, and we observed an association between highly polymorphic protease mutations and premature protease activation. These findings shed light on the diverse impacts of protease mutations and could inform future treatment strategies to either inhibit or promote protease activation.

## 2. Materials and Methods

### 2.1. Plasmids

The NL4-3 plasmid was obtained through the NIH HIV Reagent Program, Division of AIDS, NIAID, NIH: Human Immunodeficiency Virus 1 (HIV-1), Strain NL4-3 Infectious Molecular Clone (pNL4-3), ARP-114, contributed by Dr. M. Martin. The T/F panel was obtained through the NIH HIV Reagent Program, NIAID, NIH: Panel of Full-Length Transmitted/Founder (T/F) Human Immunodeficiency Virus Type 1 (HIV-1) Infectious Molecular Clones, HRP-11919, contributed by Dr. John C. Kappes. [33,34] The VIPER plasmid was generated previously [35]. Briefly, this process involves a VSQNYPIVQN protease cleavage site separating the mUKG-mKOκ FRET pair. It is linked to Vpr by an additional VSQNYPIVQN cleavage sequence. All modifications to NL4-3 were made using the NEB HiFi DNA assembly reagent or with overlap PCR, digestion, and ligation into the NL4-3 backbone.

### 2.2. Antiretroviral Drugs

Bictegravir (26532), delavirdine (24026), dolutegravir (22191), elvitegravir (17798), and GSK744 (27215) were obtained from Cayman Chemical. All other drugs were obtained from the NIH HIV Reagent Program, NIAID: Amprenavir, ARP-8148; Darunavir (Prezista, TMC 114), ARP-11447; Indinavir sulfate, ARP-8145; Lopinavir, HRP-9481; Ritonavir, ARP-4622; Saquinavir, ARP-4658; Tipranavir (Aptivus), ARP-11285; Raltegravir, HRP-11680; Efavirenz, HRP-4624; Lamivudine (3TC), HRP-8146; and Tenofovir, HRP-10199, contributed by DAIDS/NIAID.

### 2.3. Virus Production

HEK293T cells were cultured in DMEM containing 10% FBS with 1% penicillin–streptomycin. The virus was produced via the PEI transfection of HEK293T cells one day after they were plated, with viral DNA added in a 3:1 ratio with VIPER-Vpr DNA. A total of 5 µM of each inhibitor was added, where indicated, at the time of transfection. Virus was harvested 48 h post-transfection, and the supernatant was either fixed in 4% paraformaldehyde for analysis using nanoscale flow cytometry or used for infection or Western blot assay. Prior to Western blotting, virus was concentrated by centrifuging at 30,000 RPM (109,000 RCF) for 90 min through a 20% sucrose cushion and resuspended in PBS.

### 2.4. Nanoscale Flow Cytometry

#### 2.4.1. Biosafety

All viruses were fixed with 4% paraformaldehyde prior to analysis to lower the risk of analyzing infectious HIV-1 particles. In addition, the FACSAria instrument used for analysis was equipped with a negative-pressure BioBubble (Propel Labs) containing a 0.3 µm HEPA filtration system with a 200 cfm airflow. The system integrity is regularly maintained by the Case Western cytometry core using a “Glo-germ” protocol. All equipment and procedures complied with the proposed NIH/International Society for the Advancement of Cytometry standards and were approved by the Case Western Reserve University Environmental Health Safety office.

#### 2.4.2. Analysis

Analysis was performed using a FACSAria II SORP sorter (Becton Dickinson, Franklin Lakes, NJ, USA). Thresholding was set using a 150 mW 532 nm laser, which detects mKOκ positivity using a 575/26 filter. A non-fluorescent PBS sample was used as a negative control, and the mKOκ channel voltage was increased until less than 1 event/second was detected. The processing signal in recorded virions was based on increased mUKG positivity, detected with a 100 mW 488 nm laser and 515/20 filter, and a decreased unprocessed FRET signal, detected with the 488 nm laser and 575/40 filter. Sheath fluid was filtered with a 0.2 µm pore, and viruses were diluted to collect approximately 6000 events per second or less to avoid coincidental swarm detection, as previously described [36].

### 2.5. Infection Assay

JLTRG-R5 cells were stably transfected with an LTR-GFP construct that expresses EGFP as a reporter of HIV-1 infection. The LTR (long terminal repeat) in this case is the HIV-1 promoter that requires the HIV-1 Tat protein to promote gene expression. Cells were obtained through the NIH AIDS Reagent Program, Division of AIDS, NIAID, NIH: Jurkat LTR-GFP CCR5+ cells (JLTRG-R5) (Cat #11586) provided by Dr. Olaf Kutsch. JLTRG-R5 cells express the CD4 receptor and both CCR5 and CXCR4 coreceptors to mediate HIV-1 infection. Cells were cultured in RPMI supplemented with 10% FBS and 1× penicillin–streptomycin. A total of 1 × 10^5^ cells were aliquoted in a v-bottom 96-well plate before virus was added for infection. A total of 5 µM of each inhibitor was also added, where indicated. The plate was spinoculated at 1200 g for 2 h to assist infection before transferring the cells to a flat-bottom plate and incubating them at 37 °C [37]. Two days post-infection, cells were analyzed for productive infection based on EGFP expression. Cells were fixed with a 1% paraformaldehyde solution. Analysis was performed with a BD LSR II flow cytometer.

### 2.6. Western Blot

Virus was diluted in 4× Laemmli buffer (BioRad 1610747) containing β-mercaptoethanol and boiled for 5–10 min at 95 °C. Samples were loaded on a 4–15% SDS gel with a Precision Plus dual-color ladder and processed for approximately 1.5 h at 80 V (BioRad 4561086DC). Gels were transferred using the BioRad Trans-Blot system onto a nitrocellulose membrane (BioRad 1704158). The membranes were stained using the Thermo Fisher (Waltham, MA, USA) iBind system with either an anti-capsid or anti-protease primary antibody, which were obtained through the NIH HIV Reagent Program, Division of AIDS, NIAID, NIH; the antibodies were Anti-Human Immunodeficiency Virus 1 (HIV-1) CA Gag Monoclonal (#24-2), ARP-6457, contributed by Dr. Michael Malim, and Anti-HIV-1 Protease Polyclonal, ARP-4105 (no longer available). An anti-mouse (BioRad 170-6516) or anti-rabbit (Dako P021702-2) HRP-conjugated secondary antibody was used, respectively. HRP was detected using Clarity Western ECL substrate (BioRad 170-5061). Imaging and densitometry analysis were performed using Thermo Fisher iBright.

## 3. Results

### 3.1. The VIPER-Vpr Nanoscale Flow Cytometry Assay Can Simultaneously Quantify Protease Activity and Particle Production

**Figure 1:**
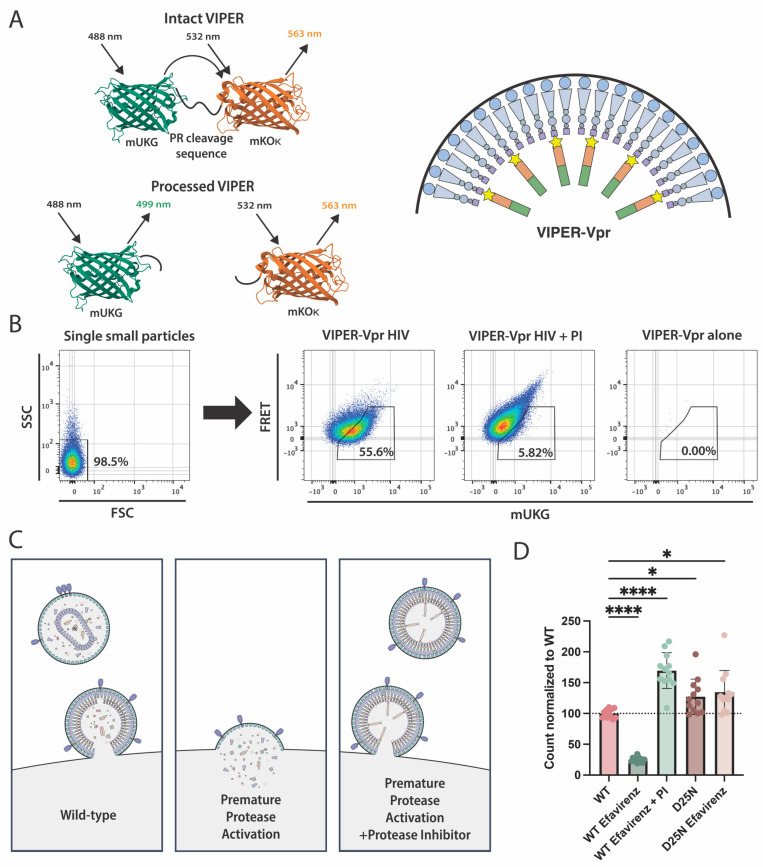
The VIPER-Vpr assay used to study protease activation. (**A**) Schematic of VIPER-Vpr protease reporter. VIPER consists of the fluorescent proteins mUKG and mKOκ, separated by the protease cleavage sequence VSQNYPIVQN. When the reporter is intact, excitation of mUKG results in FRET and the excitation of the nearby mKOκ protein. When protease processes the reporter, FRET is disrupted. VIPER (green and orange rectangles) is linked to the viral protein Vpr (star symbol) that non-covalently binds to the viral Gag p6 (purple square symbol) protein and is specifically incorporated into budding viral particles. Representative fluorescent protein structures were generated using GFP from the RCSB PDB (RCSB.org) of PDB ID 2QLE [39,40]. (**B**) Sample nanoscale flow cytometry data on HIV-1 labeled with VIPER-Vpr. The cytometer threshold was set based on mKOκ fluorescence, and the mKOκ channel voltage was adjusted such that only mKOκ-positive particles were recorded. When co-expressed with HIV-1, VIPER-Vpr is incorporated into viral particles. Individual small particles are first gated using forward and side scatter. VIPER processing is then gated as shown, based on increased mUKG fluorescence and decreased FRET. Wild-type HIV-1 resulted in 55.8% processing. Inclusion of a protease inhibitor (PI) like 5 µM darunavir prevents VIPER from being processed and is used as a negative control for gating (6.28% processed events). Detection of VIPER-positive events in the absence of HIV-1, i.e., the packaging of VIPER into extracellular vesicles, is minimal, with less than 1 event/second recorded (0.00% processed events). (**C**) Relationship between premature protease activation and particle production. Premature protease activation causes the number of particles produced to decrease. The addition of a protease inhibitor counteracts protease activation and restores efficient viral production. (**D**) Analysis of premature protease activation with VIPER-Vpr. NL4-3 virions labelled with VIPER-Vpr were recorded using nanoscale flow cytometry. The cytometer was set up as depicted in Figure 1B, with thresholding set to only record mKOκ-positive events with minimal detection of non-specifically labelled extracellular vesicles. The relative number of particles detected in 20 s corresponds to the relative concentration of viruses in the sample. Applying 5 µM of efavirenz resulted in a decrease in the number of particles produced, but the addition of 5 µM protease inhibitor or the protease D25 catalytic site mutation restored particle production. The data shown are the mean values from twelve biological replicates with the standard error of the mean. A one-way Brown–Forsythe and Welch ANOVA with Dunnett’s T3 multiple comparison statistical analysis was performed. *p*-values are denoted as * (*p* < 0.05) and **** (*p* < 0.0001).

Our lab has developed a unique nanoscale flow cytometry-based assay to study protease function in individual and intact virions [35]. This assay makes use of a fluorescence-based reporter system we refer to as VIPER (VIral ProteasE Reporter). VIPER consists of an mUKG (mUmikinoko-Green) and mKOκ (mKusabira-Orange-κ) FRET pair that is separated by an interchangeable protease cleavage sequence (Figure 1A). In this study, the cleavage sequence is VSQNYPIVQN, which is the natural substrate found between the matrix (MA) and capsid (CA) proteins in Gag. The HIV-1 protein Vpr is linked to VIPER to allow its specific incorporation into viral particles, as Vpr noncovalently associates with Gag p6 during viral assembly [38].

The minimum cytometer threshold was set based on the detection of mKOκ fluorescence, so only viral particles labeled with VIPER-Vpr were recorded, and the mKOκ voltage was increased to prevent dimly fluorescent, non-specifically labeled extracellular vesicles from being recorded (Figure 1B). Protease activity within individual virions was monitored using nanoscale flow cytometry, with the apparent loss of FRET and increase in mUKG fluorescence indicating the reporter was processed by protease. A low level of background processing signal was allowed when protease activity was inhibited to maximize signal resolution. In addition to the processing of VIPER, we determined the relative viral concentration based on the number of viral particles detected by the cytometer over the course of 20 s [36]. This allowed us to simultaneously monitor the efficiency of particle production and protease activity using nanoscale flow cytometry.

### 3.2. Assessing Premature Protease Activation Using Nanoscale Flow Cytometry

Premature protease activation is a developing area of investigation because of its potential ability to specifically kill infected cells [31,32]. We therefore adapted our VIPER assay to also quantify premature protease activation. Viral budding is prevented by premature protease activation, but adding a protease inhibitor abolishes protease activity and can restore efficient particle production (Figure 1C). 

We were able to observe this using the reverse transcriptase inhibitor efavirenz, which promotes premature protease activation by facilitating protease dimerization [31,32]. Efavirenz caused the production of the HIV-1 NL4-3 strain to significantly decrease, but particle production was restored when the protease inhibitor saquinavir was added (Figure 1D). Similarly, inactivating protease with a catalytic site D25N mutation also increased particle production. Inhibiting protease activity actually increased particle counts above the untreated wild-type levels, suggesting that wild-type NL4-3 exhibits a degree of premature protease activation.

### 3.3. Antiretroviral Inhibitors Have Distinctive Effects on Protease Function and Particle Production

**Figure 2:**
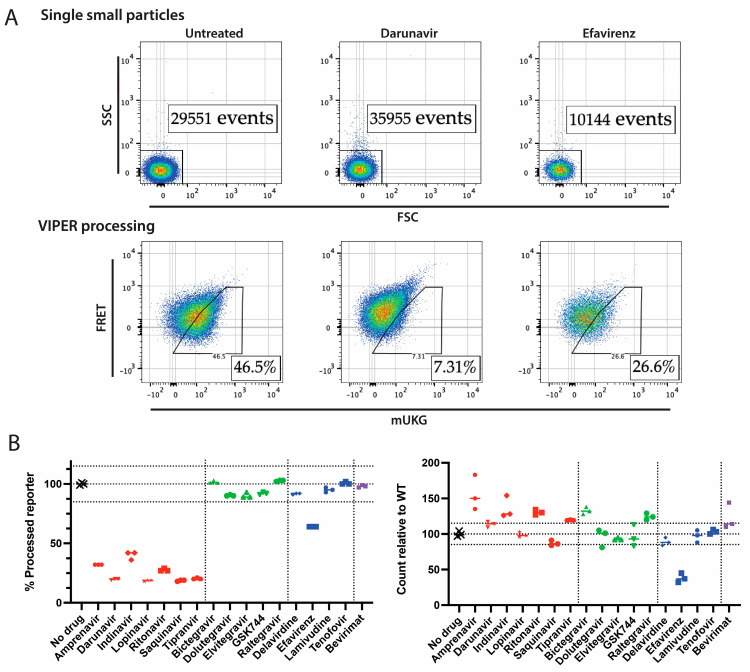
Screening of antiretrovirals for their impact on protease activity and particle production of NL4-3. (**A**) Sample nanoscale flow cytometry data. An untreated sample and darunavir- and efavirenz-treated samples are shown. The first row shows events recorded in 20 s, gated on individual events based on forward and side scatter. The particle count within each gate is shown. Subsequently, samples were plotted for VIPER processing using FRET and mUKG fluorescence. The percentage of viruses within each gate is shown. (**B**) Impact of antiretrovirals on NL4-3. An array of protease (red), integrase (green), reverse transcriptase (blue), and maturation (purple) inhibitors at a 5 µM concentration were assessed using the VIPER-Vpr assay and the NL4-3 virus. The data shown were obtained from three biological replicates and their means. The no drug, 15%-above, and 15%-below means are marked by dotted lines. The relative count and processing of VIPER were normalized to the “No drug” control.

We first assessed the utility of VIPER by testing a panel of known protease, integrase, reverse transcriptase, and maturation inhibitors. NL4-3 was produced in the presence of various inhibitors. The processing of VIPER in virions and the relative production of viral particles were measured using nanoscale flow cytometry (Figure 2A,B). Allowing for a low-level background processing signal, all of the protease inhibitors reduced VIPER processing by 60% to 82%. Most other drugs altered processing by less than 10%, with the exception of the reverse transcriptase inhibitor efavirenz, which reduced processing by 36%. This is counterintuitive considering efavirenz enhances premature protease activation. However, protease activation within the cytosol has been reported to impair the packaging of protease into released virions, and this may have had a negative impact on the processing of VIPER [41].

Protease inhibitors generally increased the rate of particle production, consistent with them presumably reducing premature protease activation. The two integrase inhibitors bictegravir and raltegravir also appeared to modestly enhance the number of particles produced. However, efavirenz significantly reduced the number of particles detected, consistent with its ability to promote premature protease activation [32].

**Figure 3:**
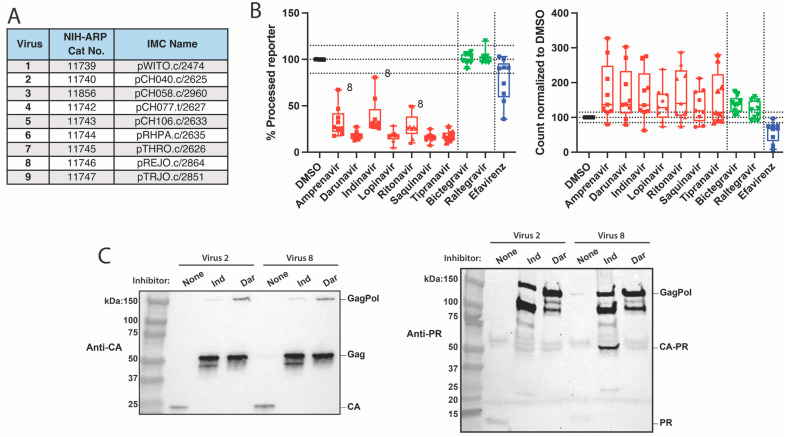
Screening antiretrovirals for their impact on protease activity and the particle production of patient-derived viruses. (**A**) Panel of transmitted/founder (T/F) viruses. The corresponding National Institutes of Health (NIH) AIDS Reagent Program (ARP) catalog numbers and infectious molecular clone (IMC) names are listed. (**B**) Impact of antiretrovirals on a panel of diverse T/F viruses. A subset of protease (red), integrase (green), and reverse transcriptase (blue) inhibitors at a 5 µM concentration were assessed in T/F viruses by using the VIPER-Vpr assay. Changes in VIPER processing and particle count, relative to a DMSO-treated control, are shown in a box-and-whisker plot. Data points for each virus represent the mean values from three biological replicates. Specific data points corresponding to virus 8 are labeled. The no drug, 15%-above, and 15%-below means are indicated with dotted lines for particle count. (**C**) Assessing the drug resistance of T/F virus 8. The T/F viruses 2 and 8 were produced in the presence and absence of the protease inhibitors indinavir (Ind) and darunavir (Dar). The Western blot analysis of capsid (CA) and protease (PR) are shown. CA-PR indicates the GagPol-derived polyprotein starting from capsid and ending with protease.

This experiment was repeated with a panel of nine patient-derived transmitted/founder (T/F) viruses instead of NL4-3 (Figure 3A) [33,34]. With this diverse set of viruses, the general trends observed with NL4-3 were replicated, although with a much greater degree of variability (Figure 3B). In particular, virus 8 (pREJO.c/2864) was consistently the least sensitive to the inhibition of processing by the protease inhibitors amprenavir, indinavir, and ritonavir. We compared the processing activity of virus 8 to the drug-sensitive virus 2 (Figure 3C). Although the processing of the Gag polyprotein did not indicate virus 8 was resistant to indinavir, the processing of GagPol did show some resistance, with the appearance of an approximately 50 kDa processing intermediate. The molecular weight of this protein corresponds to the processing of the N-terminal matrix–capsid (MA|CA) cleavage site and the site between protease and reverse transcriptase (PR|RT). Of note, MA|CA is the same cleavage sequence we use in VIPER.

There was also variability in particle production when the viruses were treated with inhibitors (Figure 3B). Although efavirenz efficiently inhibited NL4-3 particle production, there were several T/F viruses that were not as sensitive to efavirenz-induced premature protease activation. At least two of the T/F viruses, 3 and 4, had transmitted drug resistance mutations that could interfere with efavirenz binding [20]. This reveals the variability in how various resistance and polymorphic mutations can impact protease function and premature protease activation.

### 3.4. Polymorphic Hinge Mutations Can Alter Rates of Premature Protease Activation

**Figure 4:**
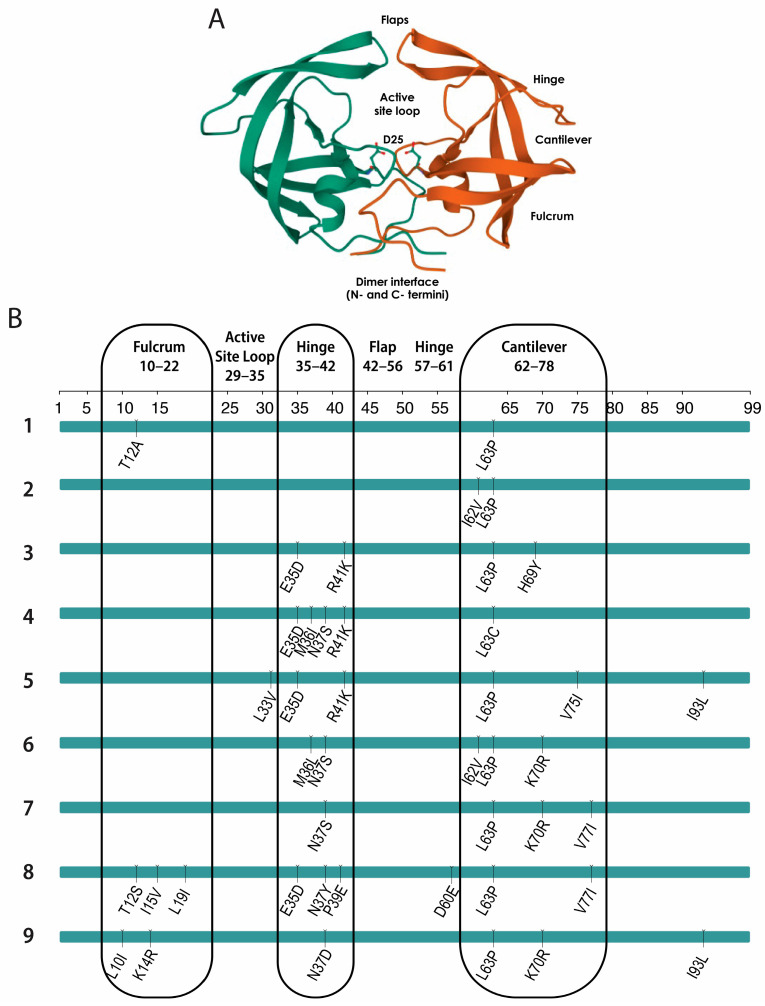
Locations of T/F protease polymorphisms. (**A**) HIV-1 protease structure. The individual domains of the HIV-1 protease dimer are labeled, including the catalytic D25 residue and the dimerization interface. The HIV-1 protease structure was obtained from the RCSB PDB (RCSB.org), available under the PDB ID 2HB4 [40,42]. (**B**) Protease mutations in a panel of T/F viruses. The protease sequences of the T/F viruses were compared to a consensus HIV-1 subtype B sequence using the Stanford HIV Drug Resistance Database HIVseq program [43,44]. Corresponding domains in the protease structure are indicated.

Using the same panel of patient-derived viruses, we sought to identify the cause of the apparent differences in premature protease activation. Because the viruses have polymorphic mutations throughout their genomes, we specifically looked at differences in the protease gene by subcloning protease into a standard NL4-3 backbone, creating NL-T/F chimeras. Each protease gene had between two and nine polymorphisms compared to a consensus HIV-1 subtype B sequence, with the majority of polymorphisms occurring in the hinge and cantilever domains of protease (Figure 4A,B).

**Figure 5:**
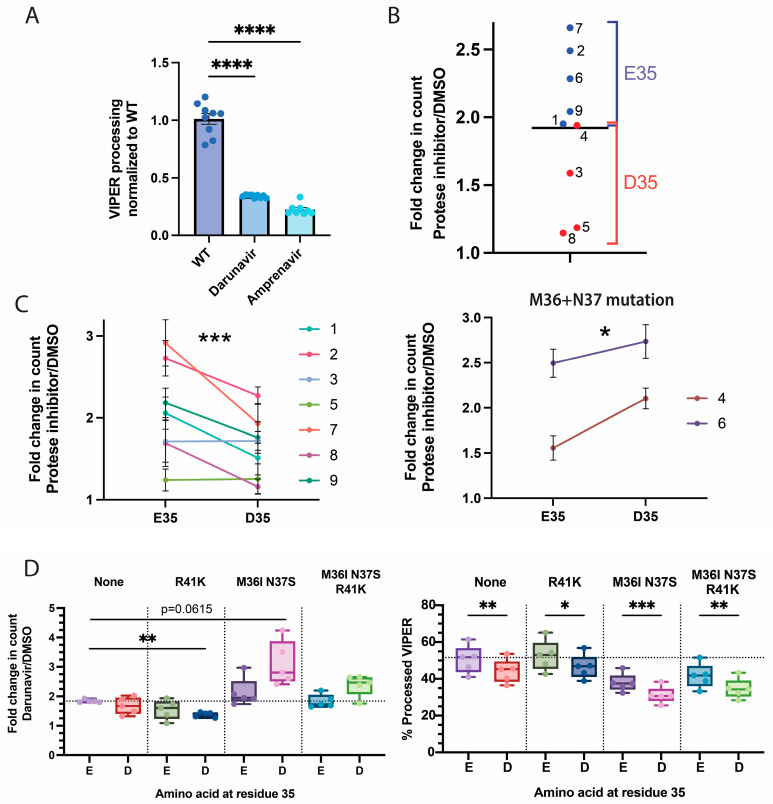
Impact of hinge polymorphisms on protease activity. (**A**) Sensitivity of NL-T/F chimera viruses to 5 µM of darunavir and amprenavir. The NL-T/F protease chimeras were tested using VIPER-Vpr in the presence or absence of 5 µM of the protease inhibitors darunavir or amprenavir. Differences in VIPER processing are shown, normalized for the untreated wild-type control. Virus 8 was excluded from the analysis of amprenavir as it appeared resistant to the drug. Each virus was tested in duplicate, and a one-way Brown–Forsythe and Welch ANOVA with Dunnett’s T3 multiple comparison statistical analysis was performed. The means and the standard errors of the means are shown. *p*-values are denoted as **** (*p* < 0.0001). (**B**) Impact of the protease 35 polymorphism on the premature protease activation of NL-T/F chimeras. The ratio of particle counts in the presence and absence of 5 µM of protease inhibitors was tested for each chimera. Viruses with E35 are presented in blue, and viruses with D35 are presented in red. Data points are the means of 4 biological replicates. The overall mean is denoted by a black line. (**C**) Assessment of the relative impact of E35 vs. D35 on premature protease activation. Residue 35 of each NL-T/F chimera was either mutated from E to D or vice versa, and the ratio of particle counts in the presence and absence of 5 µM of protease inhibitors was tested. Viruses with both M36 and N37 mutations are plotted separately. Data points represent the mean of 5 biological replicates, and the standard errors of the means are shown. An ordinary two-way ANOVA statistical analysis was performed, and *p*-values are denoted as * (*p* < 0.05) and *** (*p* < 0.001). (**D**) Relationship between multiple protease hinge polymorphisms and premature protease activation. The NL4-3 virus was mutated with the indicated M36I, N37S, and/or R41K polymorphisms, and the impact of the E35 vs. D35 polymorphism was tested. The processing of VIPER and the ratio of particle counts in the presence and absence of protease inhibitors from 5 biological replicates are shown in a box-and-whisker plot. A one-way ANOVA with Geisser–Greenhousse correction and Dunnett’s T3 multiple comparison statistical analysis was performed, and *p*-values are written or denoted as * (*p* < 0.05), ** (*p* < 0.01), and *** (*p* < 0.001).

We compared premature protease activation using either the protease inhibitors darunavir, to which all tested viruses were sensitive, or amprenavir, to which only virus 8 was resistant (Figure 5A). Using nanoscale flow cytometry, premature protease activation was estimated based on the ratio of viral events recorded within 20 s in the presence or absence of a protease inhibitor. In the absence of a drug, viruses that have high levels of premature protease activation will have impaired particle production. However, particle production will increase when a protease inhibitor is added, as illustrated in Figure 1B. Therefore, the ratio of particle counts in the presence and absence of an inhibitor will be higher with increased rates of premature protease activation.

When comparing the sequences of each virus, we found that the viruses with the lowest levels of premature protease activation shared the E35D polymorphism (Figure 5B). E35 is in the hinge region of protease and regulates the opening and closing of the protease flaps. The flaps are responsible for holding and positioning substrates next to the protease catalytic site. In subtype B viruses, E35D is very common, occurring in 32% of sequences [20,21]. D35 is the consensus sequence of the HIV-1 subtypes A, F, G, and AE [20,21].

We further investigated the role of residue 35 by mutating it in each chimera, either from E35 to D35 or vice versa. In some cases, the presence of D35 reduced the rate of premature protease activation (Figure 5C). This was true for viruses 1, 2, 7, and 9, which had no hinge mutations other than a sole N37S/D polymorphism. However, viruses encoding multiple hinge polymorphisms, including M36I/L, N37S/Y, P39E, and/or R41K, exhibited no changes or enhanced premature protease activation when combined with E35D. Viruses 4 and 6 in particular had mutations at both M36 and N37, and the addition of D35 significantly increased premature protease activation.

Of the subtype B viruses with E35D, 80.6% also have an M36, N37, or R41 mutation [20,21]. The R41K mutation is the most common, occurring in 43% of sequences with E35D [20,21]. We tested several combinations of the E35D, M36I, N37S, and R41K mutations in the NL4-3 viral strain. M36I and N37S were tested together to limit the number of constructs generated. We found that the combination of E35D and R41K significantly reduced premature protease activation (Figure 5D). In contrast, combining E35D with the M36I and N37S mutations enhanced premature protease activity. In all cases, E35D reduced the processing efficiency of VIPER, with the combination of E35D, M36I, and N37S causing the greatest reduction. This reduction in VIPER processing is consistent with reports that E35D increases protease flap flexibility and reduces substrate affinity [45,46].

### 3.5. The Impact of Major Mutations on Protease Activation and Viral Replicative Fitness

**Figure 6:**
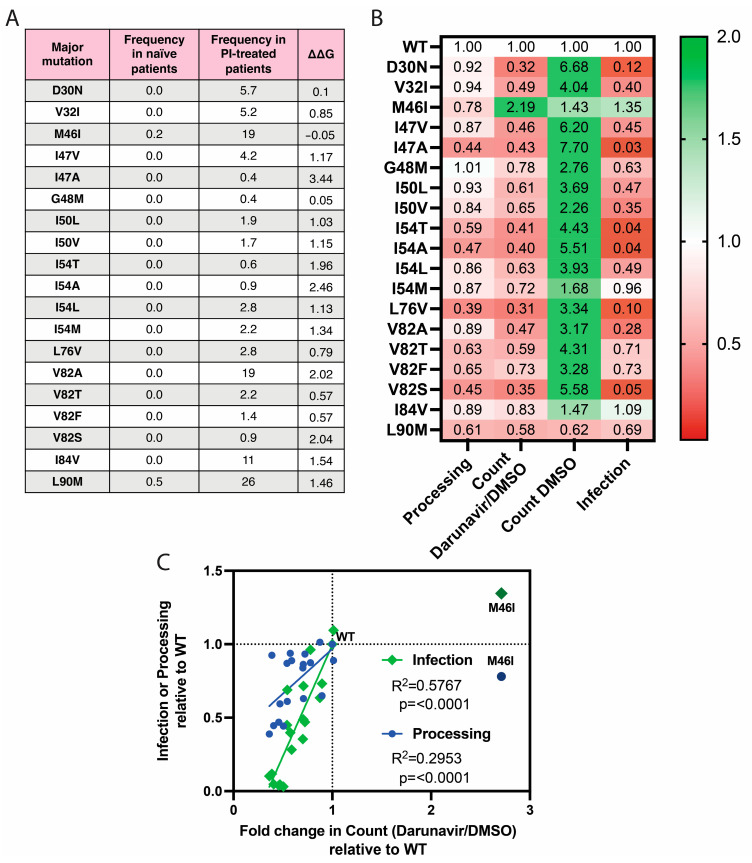
Impact of major resistance mutations on protease function and viral infectivity. (**A**) Frequency of major protease mutations in patients before (naïve) and after treatment with protease inhibitors (PI). Frequencies were obtained from the Stanford HIV Resistance database. 67,567 subtype B patient sequences before treatment and 23,458 after treatment were included, and frequencies are shown as percentages. The stability changes were estimated using the PoPMuSiC web server and the RCSB 1KJ4 protease structure [40,47,48,49]. (**B**) Impact of major protease resistance mutations on protease activity and infectivity. The detection of VIPER processing and the determination of the ratio of particle counts in the presence and absence of 5 µM of darunavir were executed using nanoscale flow cytometry. Productive infection was determined using JLTRG-R5 cells. Infectivity was normalized based on the relative particle concentration determined via nanoscale flow cytometry. Values are summarized in a heat map relative to the wild-type virus lacking resistance mutations. The data were calculated using the mean values of three biological replicates. (**C**) Correlation between the ratio of particle counts in the presence and absence of 5 µM of darunavir, VIPER processing, and infectivity. The values from Figure 5B were plotted, and the R^2^ and *p*-values were calculated using a simple linear regression. M46I was an outlier and thus excluded from the analysis.

Major resistance mutations are exceedingly rare in treatment-naive patients, but they become more frequent following treatment with protease inhibitors (Figure 6A). Generally, major mutations are predicted to destabilize the protease structure and interfere with inhibitor binding. Changes in thermodynamic stability were estimated using the PoPMuSiC web server, which predicts protein stability changes caused by single-site mutations [47]. The RCSB Protein Data Bank was used to obtain an HIV-1 protease structure for testing: 1KJ4 is a protease when bound to its MA|CA substrate, which is identical to the cleavage sequence found in VIPER [40,48,49].

We tested a panel of major resistance mutations for their impacts on protease activity. Viruses were labeled with VIPER-Vpr, and nanoscale flow cytometry was used to detect VIPER processing. Relative premature protease activation was estimated as the ratio of particle counts recorded in 20 s in the presence or absence of darunavir, as was conducted in Figure 5. Equivalent volumes of virus from the supernatant of transfected HEK293T cells were added to JLTRG-R5 cells to assess infectivity using an HIV-specific promoter to drive GFP expression. Infectivity was normalized based on the relative concentration of virions, which was assessed using the number of particles detected in 20 s using nanoscale flow cytometry. Most mutations increased particle counts, so infectivity was decreased in proportion to concentration. All data were normalized to the results when using the wild-type NL4-3 virus.

In agreement with our expectations, we found that nearly all major mutations reduced the processing of VIPER, inhibited premature protease activation, and interfered with productive infection (Figure 6B). The primary exception was M46I, which was the only mutation that appeared to enhance premature protease activation. With the exception of M46I, there was a significant correlation between the rates of premature protease activation and both VIPER processing and productive infection (Figure 6C).

### 3.6. The Impact of Accessory Mutations on Protease Activation and Viral Replicative Fitness

**Figure 7:**
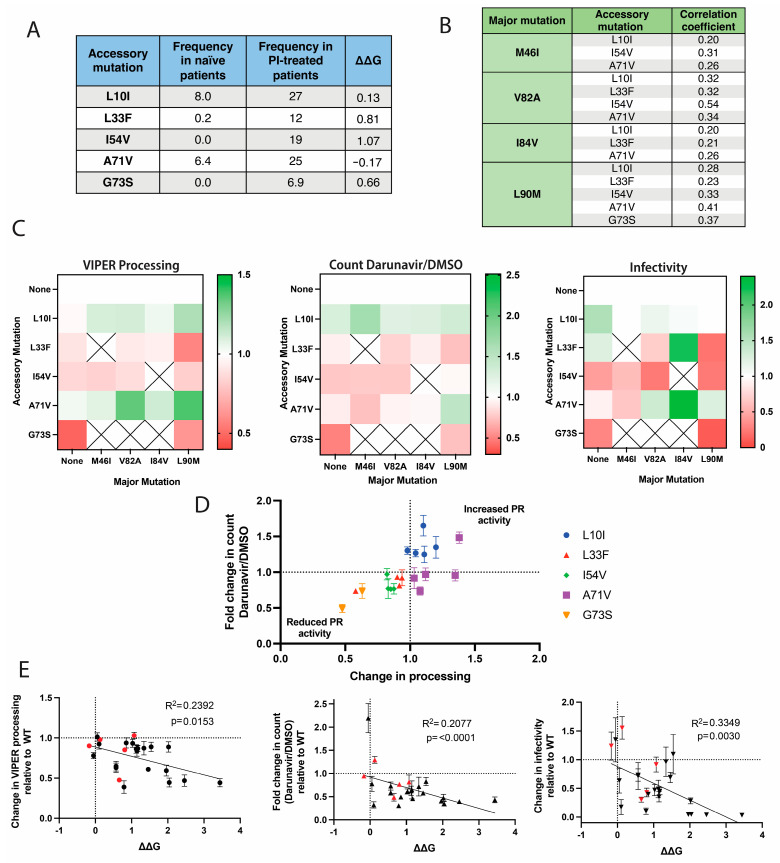
Impact of accessory resistance mutations on protease function and viral infectivity. (**A**) The frequency of accessory protease mutations in patients before (naïve) and after treatment with protease inhibitors (PI). Frequencies were obtained from the Stanford HIV Resistance database. A total of 67,567 subtype B patient sequences before treatment and 23,458 after treatment were included, and frequencies are shown as percentages. The stability changes were estimated using the PoPMuSiC web server and the RCSB 1KJ4 protease structure [40,47,48,49]. (**B**) Correlations of major and accessory mutation pairs. Correlation values were generated by Weikl and Hemmateenejad after 26,838 PI-treated patient sequences were assessed [24]. The correlation can range from −1 to 1, with values above 0 being positively correlated. (**C**) Impact of accessory protease resistance mutations on protease activity and infectivity. The combinations of major and accessory resistance mutations shown in Figure 6B were tested. The assessment of VIPER processing and the determination of the ratio of particle counts in the presence and absence of 5 µM darunavir were executed using nanoscale flow cytometry. Productive infection was determined using JLTRG-R5 cells, and infectivity was normalized based on relative particle concentration determined via nanoscale flow cytometry. Values are summarized in a heat map normalized to virus lacking accessory resistance mutations. The white crossed squares indicate combinations that were not tested. The data were calculated using the mean values of three biological replicates. (**D**) The relationship between premature protease activation and processing activity of accessory mutations paired with major mutations. The major and accessory mutation combinations from Figure 6B were plotted to compare changes in processing and the ratio of particle counts in the presence and absence of 5 µM darunavir. Values were normalized to results with the major mutation alone. Data were collected as three independent replicates, and the standard error of the mean is shown. (**E**) Correlation between protease stability and protease activity in response to resistance mutations. The average changes in VIPER processing, infectivity, and the ratio of particle counts in the presence and absence of 5 µM of darunavir were plotted against the calculated change in protease stability caused by each mutation. Accessory mutations are shown in red. A simple linear regression analysis was performed, and the R^2^ and *p*-values are shown.

As with major mutations, accessory mutations are more common following treatment with protease inhibitors (Figure 7A). In many cases, accessory mutations mitigate the destabilizing effects of major mutations, but they may also contribute to resistance when combined with major mutations. We tested combinations of the most frequent major and accessory resistance mutations (Figure 7B). L10I and A71V are relatively common in treatment-naïve patients and are two of the rare polymorphisms that are also classified as resistance mutations [20,21]. Unsurprisingly, L10I and A71V were also the most beneficial accessory mutations in terms of enhancing protease activity and infectivity (Figure 7C,D). The other accessory mutations, L33F, I54V, and G73S, were on the opposite end of the spectrum and generally impaired protease activity, although L33F did increase infectivity in some pairings (Figure 7C). 

When assessing all of the major and accessory mutations together, we found a correlation between their predicted effect on protease stability and their ability to process VIPER and facilitate premature protease activation as well as their susceptibility to infection (Figure 7E).

## 4. Discussion

The HIV-1 protease is an important target for antiretroviral therapy. The purpose of this study was to examine how polymorphic and resistance mutations regulate protease function and viral fitness. Emphasis was placed on the impact mutations could have on the feasibility of using premature protease activators as an HIV-1 cure strategy.

**Figure 8:**
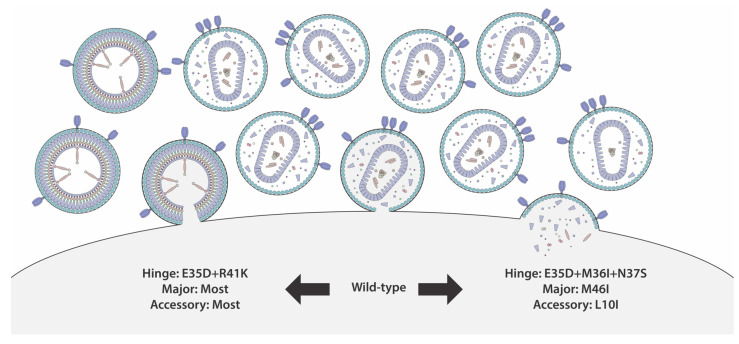
Summary of key findings. The combined hinge polymorphisms E35D+R41K and most major and accessory protease inhibitor resistance mutations impair the rate of premature protease activation and potentially delay activation kinetics. On the other hand, premature protease activation is promoted by the combined E35D+M36I+N37S mutations and the resistance mutations M46I and L10I. This process can inhibit particle production and increase the risk of the production of defective particles that lack essential proteins [41].

We assessed the impact of major and accessory resistance mutations on protease activity using VIPER and correlated their predicted impact on protease stability with protease function. M46I was the only major mutation predicted to stabilize protease structure, and it was also able to considerably enhance infectivity and promote premature protease activation (Figure 8). This is consistent with the results of an in vitro growth competition assay that showed that the M46I mutant and wild-type virus were equally fit [50]. In addition, in a clinical study, M46I was found to increase the rate of viral transmission, suggesting it provides a replicative advantage [51]. 

The L10I accessory mutation also enhanced protease activity and replication, consistent with its reported ability to stabilize the structure of protease [23,52]. Similar reports have been made concerning the A71V accessory mutation [23,53]. Although the G73S mutation is reported to be able to stabilize the protease dimer, its catalytic efficiency drastically changes depending on the substrate, with some substrates being processed more than 80% less efficiently by G73S than by wild-type protease [54]. This could certainly lead to the significantly impaired VIPER processing and infectivity that we observed.

Polymorphic mutations are typically not classified as resistance mutations, although there are a few exceptions, including the aforementioned L10I and A71V mutations, both of which are capable of enhancing protease activity. Though not classified as resistance mutations, several other polymorphisms have been associated with drug resistance and poor virological response [46,55,56,57,58]. In a small study looking at the 16 most common protease polymorphisms, the presence of 5 or more polymorphisms significantly predicted worse treatment outcomes [58]. We assessed several hinge polymorphisms that appeared to regulate protease function. We observed that E35D was associated with reduced premature protease activation and significantly impaired processing of VIPER. This is consistent with the reported ability of E35D to increase the flexibility of the protease flaps and promote a more “open” configuration, which is detrimental to substrate binding [45,46]. This “open” flap configuration is a common mechanism also used by resistance mutations to interfere with the binding of protease to inhibitors [56]. 

The nearby M36I polymorphism is also reported to increase protease flexibility and is associated with protease inhibitor resistance [59]. However, we observed that M36I, in the presence of N37S, enhanced premature protease activation when combined with E35D. Similar to G73S, M36I has reduced affinity for many substrates but enhanced affinity for others [57,60]. This may account for the unexpected result that M36I reduces the processing of VIPER but appears to enhance premature protease activation.

For simplicity, we only tested one major protease mutation with one accessory protease mutation in the absence of an inhibitor. However, mutations outside of protease can also contribute to drug resistance, including those in and around the Gag cleavage sites, which are reported to enhance the efficiency with which these sites are processed [61,62,63,64]. The impact of resistance mutations also depends on which other mutations are present and to which substrate or inhibitor the protease is bound [24,48,54,65,66]. Therefore, as resistance mutations accumulate, there may be drastic differences in how these mutations manipulate protease function.

Inducing premature protease activation is a novel method of specifically killing HIV-1-infected cells. This exciting prospect has sparked interest in characterizing the feasibility of this potential new therapeutic strategy [29,30,31]. Existing non-nucleoside reverse transcriptase inhibitors (NNRTIs) can promote premature protease activation, but they are only effective in high concentrations in vitro [31,32]. If NNRTIs are used as a scaffold to design more effective premature protease activators, it will be important to ensure there is no cross-resistance with existing NNRTIs. A study including 47,215 people in the United States found that 12.0% had transmitted drug resistance for a non-nucleoside reverse transcriptase inhibitor [67]. Of the nine T/F viruses we tested, two had mutations associated with moderate-to-high resistance to efavirenz, which reflected in the inability of efavirenz to efficiently induce premature protease activation in these viruses. In addition, our results suggest that the presence of protease inhibitor resistance mutations and common polymorphisms can significantly influence protease activity, making protease more or less likely to prematurely activate. There will likely be considerable variability in the susceptibility of primary HIV-1 isolates to premature protease induction that could hinder drug development, and, given the ease with which polymorphic residues can mutate, drug resistance will need to be carefully monitored. 

The VIPER reporter assay is a powerful tool for simultaneously screening the characteristics of protease activity and particle production. However, there are certainly limitations to consider. The detection of virions requires that VIPER be incorporated into virions via interactions between the conjugated Vpr and Gag. The strength of these interactions may vary between different Gag sequences, which would compromise this assay’s reliability with respect to certain patient viruses. In addition, we used VIPER to report on the ability of protease to process the encoded VSQNYPIVQN substrate. Although this cleavage sequence can be modified, the assay cannot fully encapsulate the impact mutations have on protease activity, which varies depending on the specific substrate. This means that the processing of VIPER can occur despite impaired processing of Gag and diminished infectivity. We observed this with the T/F virus 8 when treated with indinavir: despite efficient processing of VIPER and modest processing of GagPol, there was no improvement in Gag processing compared to a fully sensitive virus. Finally, we used the ratio of particle counts in the presence and absence of a protease inhibitor to estimate premature protease activity. However, several factors like drug toxicity, side effects, or resistance could also impact this ratio, and these results will need additional validation.

Using nanoscale flow cytometry, we highlighted some of the potential pitfalls that could impact the design and efficacy of premature protease activators. Our data suggest that viral heterogeneity will likely pose a considerable challenge with respect to inducing premature protease activation.

## Data Availability

The raw data supporting the conclusions of this article and the MIFlowCyt-EV information are available on Zenodo.
